# Macrophages in Acute Myeloid Leukaemia: Significant Players in Therapy Resistance and Patient Outcomes

**DOI:** 10.3389/fcell.2021.692800

**Published:** 2021-06-24

**Authors:** Katerina E. Miari, Monica L. Guzman, Helen Wheadon, Mark T. S. Williams

**Affiliations:** ^1^Charles Oakley Laboratories, Department of Biological and Biomedical Sciences, Glasgow Caledonian University, Glasgow, United Kingdom; ^2^Department of Hematology & Medical Oncology, Graduate School of Medical Sciences, Cornell University, New York, NY, United States; ^3^Paul O’Gorman Leukaemia Research Centre, College of Medical, Veterinary and Life Sciences, University of Glasgow, Glasgow, United Kingdom

**Keywords:** patient outcomes, therapy resistance, BMME, M2-like macrophages, CD163^+^CD206^+^, acute myeloid leukaemia

## Abstract

Acute Myeloid Leukaemia (AML) is a commonly occurring severe haematological malignancy, with most patients exhibiting sub-optimal clinical outcomes. Therapy resistance significantly contributes towards failure of traditional and targeted treatments, disease relapse and mortality in AML patients. The mechanisms driving therapy resistance in AML are not fully understood, and approaches to overcome therapy resistance are important for curative therapies. To date, most studies have focused on therapy resistant mechanisms inherent to leukaemic cells (e.g., *TP53* mutations), overlooking to some extent, acquired mechanisms of resistance through extrinsic processes. In the bone marrow microenvironment (BMME), leukaemic cells interact with the surrounding bone resident cells, driving acquired therapy resistance in AML. Growing evidence suggests that macrophages, highly plastic immune cells present in the BMME, play a role in the pathophysiology of AML. Leukaemia-supporting macrophage subsets (CD163^+^CD206^+^) are elevated in preclinical *in vivo* models of AML and AML patients. However, the relationship between macrophages and therapy resistance in AML warrants further investigation. In this review, we correlate the potential links between macrophages, the development of therapy resistance, and patient outcomes in AML. We specifically focus on macrophage reprogramming by AML cells, macrophage-driven activation of anti-cell death pathways in AML cells, and the association between macrophage phenotypes and clinical outcomes in AML, including their potential prognostic value. Lastly, we discuss therapeutic targeting of macrophages, as a strategy to circumvent therapy resistance in AML, and discuss how emerging genomic and proteomic-based approaches can be utilised to address existing challenges in this research field.

## Introduction

Acute Myeloid Leukaemia (AML) is a genetically diverse haematological malignancy common in older adults, with 5-year survival rates less than 20% for the majority of AML patients, aged between 60 and 74 (Watts and Nimer, 2018). Importantly, survival rates have not significantly improved over the last two decades. In part, this is due to almost 70% of patients relapsing following current standard of care (Heuser et al., 2020), which has remained largely unchanged, despite extensive research into the pathophysiology of the disease. AML is a clonal disease, in which early clonal mutations in haematopoietic stem and progenitor cells (HSPCs), affecting epigenetic regulators, give rise to primitive leukaemic stem and progenitor cells (LSPCs). These cells then differentiate to early progenitors and acquire pre-leukaemic mutations prior to giving rise to leukaemic cells (Shlush et al., 2014; Desai et al., 2018). Traditionally, sub-classification and treatment stratification of AML patients was based on criteria including cytogenetic profile, ranging from chromosomal translocations associated with favourable prognosis [e.g., t(8;21)(q22;q22.1);RUNX1/RUNX1T1], to more adverse cytogenetics (e.g., rearrangements of mixed lineage leukaemia (MLL-r) gene on chromosome 11q23 and complex karyotypes). However, following recommendations from the European Leukaemia Network (Döhner et al., 2010), screening for distinct and recurrent molecular drivers of AML, including Nucleophosmin 1 (*NPM1)*, fms like tyrosine kinase 3 (*FLT3*), and CCAAT/enhancer-binding protein alpha (*CEBPA*), are currently being used in routine practice, and have significantly improved individual prognosis and clinical management through more targeted therapeutic approaches (Döhner et al., 2017). Despite this, prognosis can still remain highly variable, due to the complexity of the disease and other underlying co-morbidities relating to the age of the patients. This has led to significant progress in identifying the molecular mechanisms driving therapy resistance in AML (Konopleva et al., 2016). To a certain extent, the discovery of therapy resistance mechanisms has been catalysed *via* initial reports from the Beat AML trial, a large comprehensive study focusing on the correlation between genetic data (whole exome and RNA sequencing) and treatment sensitivity in AML (Tyner et al., 2018). The drive to find more targeted therapies to administer either alone or in combination with standard of care chemotherapy has led to several clinical trials investigating their efficacy (Supplementary Table 1). Two of these inhibitors, CYC065/Fadraciclib and AZD5991, exhibit potent AML killing properties, within *in vitro* and *in vivo* models of AML, thus representing particularly promising novel anti-AML agents. Fadraciclib is an aminopurine ATP competitive multi-cyclin-dependent kinase (CDK) inhibitor, selectively blocking the function of CDK2 and CDK9 (Saladino et al., 2015; Frame et al., 2020). Inhibition of CDK9 prevents the CDK9-driven elongation step in the transcription of short-lived proteins, including the pro-survival/anti-apoptotic protein myeloid cell leukaemia 1 (MCL-1) (Frame et al., 2020). Importantly, AML cell survival is dependent on MCL-1 expression (Kadia et al., 2019), with elevated MCL-1 levels driving intrinsic and acquired AML drug resistance (Pan et al., 2015; O’Reilly et al., 2018), and also correlating with disease relapse (Li X. X. et al., 2019).

Recent studies show that Fadraciclib elicits rapid apoptosis in a panel of AML cell lines *in vitro, via* the rapid loss of MCL-1 protein expression, and reduces leukaemia burden in xenograft models of MLL (EOL-1) and non-MLL (HL-60) AML, exhibiting superiority over the conventional AML therapeutic cytarabine/ara-C, in this setting (Frame et al., 2020).

AZD5991 is a highly selective macrocyclic MCL-1 inhibitor, displaying high affinity for MCL-1, and promoting the apoptosis of several AML cell lines, *via* caspase-dependent degradation of MCL-1 protein (Tron et al., 2018). Furthermore, AZD5991 induced tumour regression in the MV4-11 xenograft model of AML and exhibited a synergistic effect on tumour regression in the OCI-AML3 xenograft model of AML, when combined with the selective BCL-2 inhibitor venetoclax vs. venetoclax monotherapy. Recent FDA approval of two new targeted therapies, Glasdegib, a Hedgehog pathway inhibitor (Norsworthy et al., 2019) and Venetoclax, a selective BCL-2 inhibitor (Guerra et al., 2019), for elderly AML patients have been granted. This has resulted in some patients exhibiting improvement in disease, however, not all patients improve following treatment. Drug resistance therefore still represents a major reason for treatment failure, disease relapse and subsequently death in AML patients. Most studies have focused mainly on cell intrinsic mechanisms of drug resistance, overlooking to some degree extrinsic/acquired mechanisms of drug resistance. It has been long recognised that interactions of leukaemic cells with bone marrow (BM) resident cells and extracellular matrix components in their surrounding BM microenvironment (BMME) can drive therapy resistance in AML. Over the last 5 years, accumulating evidence has suggested a role for macrophages (Mφs) in the pathogenesis of AML, however, this has not been fully elucidated (Al-Matary et al., 2016; Sioud et al., 2019; Xu et al., 2019; Song et al., 2020). Here we present a comprehensive overview of: the molecular mechanisms that underlie the ability of AML blasts to re-educate the surrounding BMME and Mφs to a leukaemia supportive phenotype; the pro-survival signalling pathways potentially driving Mφ-mediated drug resistance in leukaemic cells; how particular Mφ subsets and their frequency correlate with key clinical outcomes in AML patients; the therapeutic targeting of Mφs in AML, and finally we discuss the most significant challenges that still remain in this field, and how these are beginning to be addressed *via* advanced and/or emerging technologies.

## Bone Marrow Niche Co-Option/Remodeling by AML Cells

**FIGURE 1:**
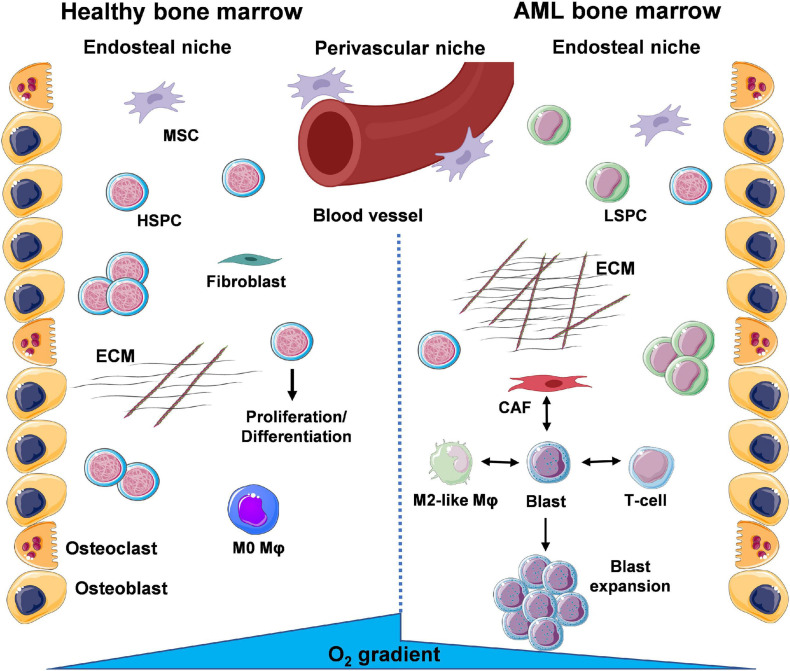
A glance at the bone marrow microenvironment (BMME) in health and in leukaemia. The bone marrow (BM) contains a number of different cell types that coordinate their functions to maintain tissue and whole-body homeostasis and support daily blood production through haematopoietic stem and progenitor cells (HSPCs). These cells exist within specific niches, distinguished by oxygen and nutrient gradients, namely the endosteal/osteoblastic niche, shaped by bone-lining osteoblasts and bone-resorbing osteoclasts, and the perivascular/vascular niche. Under basal conditions, HSPCs will migrate from the endosteal to the perivascular niche where they exit dormancy and enter the cell cycle to divide and differentiate into mature cells of the haematopoietic system. Throughout the BM, deposition of extracellular matrix (ECM) by resident fibroblasts and signalling *via* mesenchymal stem/stromal cells (MSCs) supports normal function of HSPCs. During AML, the presence of leukaemic stem and progenitor cells (LSPCs) results in expansion of the oxygen and nutrient gradients, creating an environment inhospitable to normal HSPC function. Subsequent expansion of blast populations ensures further optimal conditions for the leukaemic cells *via* cross-talk with surrounding resident cells. Interactions of leukaemic blasts with γ/δ T-cells (immune suppression), cancer-associated fibroblasts (CAFs) (enhanced ECM deposition and loss of organisation) and M2-like macrophages (Mφs) (anti-inflammatory, tumour-promoting) are the focus of a number of studies (created using Servier Medical Art; Created using Biorender.com).

The BMME can be separated into two distinct niches based on location of the cells and conditions they are exposed to (Figure 1). Bone-producing osteoblasts define the endosteal/osteoblastic niche, with reduced oxygen (hypoxia) and nutrient levels compared to the more central regions of the BM. In turn, these central regions near the vasculature form the perivascular niche, enriched in oxygen and nutrient supply. As a result, oxygen/nutrient gradients drive the formation of separate microenvironments, supporting the function of specialised cell types that cooperate to ensure homeostasis of the haematopoietic system (Nwajei and Konopleva, 2013). Within the endosteal/osteoblastic niche, normal HSPCs are capable of interacting with supportive BM stromal cells (BMSCs), including osteoblasts, adipocytes, endothelial cells, mesenchymal stromal cells (MSCs), and fibroblasts, as well as immune cells, including monocytes and Mφs. This provides HSPCs with pro-survival signals, including HSPC-to-osteoblast interactions such as C-X-C Motif Chemokine Ligand 12- C-X-C chemokine receptor type 4 (CXCL12-CXCR4), maintaining the quiescent/non-cycling pool of HSPCs (Zhou et al., 2016). Furthermore, studies show that Mφs are involved in the retention of HSPCs within the BM (Chow et al., 2011). In AML, LSPC populations exploit the environmental conditions within the BM to support their own survival. Competition for oxygen and nutrients between LSPCs and HSPCs results in expansion of hypoxic areas from the endosteal niche towards the perivascular niche (Figure 1).

The environment that arises is suboptimal to support the haematopoietic functions of HSPCs. Overall, LSPCs co-opt the HSPC niches to ensure their own survival and proliferation (Nwajei and Konopleva, 2013). Leukaemic cells have also been shown to modify their surrounding BMME, exhibiting the capacity to re-educate stromal cells to a leukaemia-supporting phenotype. In turn, AML blasts and AML-influenced stromal cells can then reprogram monocytes and Mφs to a leukaemia-supporting phenotype, *via* their respective secretomes (i.e., released microvesicles, exosomes, growth factors, cytokines, and chemokines).

BM-MSCs derived from AML patients, have been shown to release higher levels of the proinflammatory chemokine C-X-C motif ligand 8 (CXCL8) compared to BM-MSCs isolated from healthy counterparts (Cheng et al., 2019). CXCL8 can enhance the survival and proliferation of AML blasts *via* the phosphoinositide-3-kinase (PI3K)/Akt pathway. In line with these findings, Çelik et al. (2020) demonstrate considerably elevated CXCL8 levels in BM plasma samples from AML patients, compared to BM plasma from healthy individuals. Taken together these findings suggest that MSCs could represent major producers of CXCL8 in the AML BMME. Interestingly, a functionally diverse population of fibroblasts arises in the BMME either through contact-dependent effects (e.g., cancer-to-resident fibroblast or cancer-to-MSC direct cell-to-cell interactions) or contact-independent effects (via cancer secreted factors, e.g., transforming growth factor beta, TGF-β). These cells are collectively termed cancer-associated fibroblasts (CAFs). Having been extensively studied in solid cancers, CAFs are now of particular interest in blood cancers, including AML. CAFs have been shown to be present in the BM of AML patients, with elevated levels of the CAF-differentiating factor TGF-β in BM plasma derived from AML patients compared to healthy volunteers (Zhai et al., 2016). Similar to MSCs, in solid cancers, CAFs also exhibit immunomodulatory properties, as observed *via* their release of growth factors, pro-inflammatory cytokines and chemokines. These factors promote growth/expansion (e.g., macrophage-colony stimulating factor, M-CSF), recruitment (e.g., C-C motif ligand 2, CCL2), differentiation (e.g., M-CSF) and polarisation [e.g., M-CSF, interleukin 6 (IL-6), CXCL8, and TGF-β] of monocytes and Mφs, towards an M2-like phenotype (Presti et al., 2018). Evidence for this potentially occurring in AML, comes from findings showing that most of these factors are elevated in BM plasma and/or blood plasma of AML patients especially IL-6, CCL2, with a corresponding increase in M2-like CD206^+^ monocytes and M2-like CD163^+^CD206^+^ Mφs (Table 1) occurring in the BM of AML patients (Mazur et al., 2007; Mussai et al., 2013; Sanchez-Correa et al., 2013; Su et al., 2013; Al-Matary et al., 2016). It is well-established that fibroblasts and MSCs also significantly contribute towards acquired BMME-driven therapy resistance in leukaemia and in particular AML (Salman et al., 2015; Busch and Wheadon, 2019; Boutin et al., 2020). However, the impact of immunomodulation *via* BM infiltrating immune cells, such as monocytes and Mφs on drug resistance in AML, still remains largely unknown.

**TABLE 1:** List of macrophage immunophenotype markers utilised for *in vivo* and *in vitro* research.

**Immunophenotype**	**Species**	**Cell type**
***Macrophage markers***		
CD163^+^CD206^+^	Human/Murine	M2-like Mφ
CD11b^+^Ly6G^–^MHCII^–^Ly6C^–^	Murine	AAM (M2-like Mφ)—BM
Ly6C^–^CD206^–^	Murine	M2-like Mφ
CD3^–^GR1^*low*^MCSFR^*int*^F4/80^*hi*^SSC^*low*^	Murine	BM and spleen LAM populations
CD11b^–^F4/80^+^CD169^+^VCAM1^+^	Murine	BM-resident Mφ/Erythroblastic island Mφ
CD169^+^/SIGLEC1^+^	Human/Murine	BM-resident Mφ
CCR2^+^CD14^++^CD16^–^	Human	Monocyte/TAM precursor
CD11b^+^Ly6C^*low*^MHCII^*low*^	Murine	M2-like Mφ—spleen
CD11b^+^Gr1^–^F4/80^+^	Murine	TAM
***Additional commonly used markers***		
Ly6C^+^CD206^–^	Murine	M1-like Mφ
CD11b^*high*^GR1^*int*^	Murine	Non-malignant BM Mφ
CD80^+^	Human/Murine	M1-like Mφ
CD86^+^	Human/Murine	M1-like Mφ
ARG1	Murine	AAM
CHI3L1, YM1	Human, murine	Alternatively activated (M2) myeloid cell
TIE2^+^	Human/Murine	M2-like Mφ In close proximity to vasculature—Highly angiogenic activity
CD68^+^	Human/Murine	Pan-Mφ marker, CD68^+^CD163^+^ immunophenotype for detecting M2-skewing

*AAM, alternatively activated macrophage; ARG1, arginase-1; BM, bone marrow; CHI3L1 (YM1), chitinase-3-like protein 1; LAM, leukaemia-associated macrophage; Mφ, macrophage; TAM, tumour-associated macrophage.*

## Mφ Phenotypes at Steady State and in the Context of Cancer/Leukaemia

**FIGURE 2:**
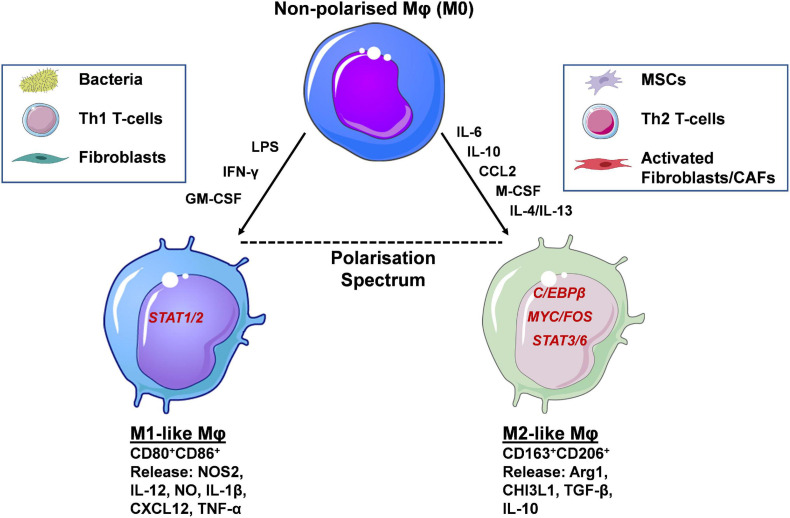
Macrophage polarisation occurs *via* activation of distinct transcription factors and determines their function. Macrophages (Mφs) are highly plastic cells, adopting phenotypic and functional states that can be classified along a polarisation spectrum. At the two opposite ends of this spectrum lie the M1-like and M2-like Mφs. The former are induced by the presence of inflammatory mediators (e.g., LPS, IFN-γ, GM-CSF), signalling through STAT1/2, and display expression of CD80 and CD86 cell surface markers. Soluble, pro-inflammatory mediators released resemble Th1-like cytokines such as IL-12, IL-1β and TNF-α. Functional polarisation towards an M1-like phenotype occurs in the presence of bacteria, Th1 T-cells and fibroblasts and their respective secreted factors. At the other end of the spectrum lie alternatively activated M2-like Mφs, induced by IL-6, IL-10, M-CSF, CCL2, and IL-4/IL-13 released by mesenchymal stem/stromal cells (MSCs), Th2 T-cells and fibroblasts/cancer associated fibroblasts (CAFs). The main transcription factors involved in M2-like polarisation are C/EBPβ, MYC/FOS, and STAT3/6. Their secretome includes a number of anti-inflammatory, Th2-like mediators, such as Arg1, TGF-β and IL-10. Cell-surface expression of CD163 and CD206 distinguish M2-like from M1-like Mφs and have the potential to be used as prognostic markers in a number of human cancers, including AML (created using Servier Medical Art; Created using Biorender.com).

Mφs exhibit a high degree of plasticity, existing within a broad spectrum/continuum of different polarisation and functional states (Murray et al., 2014). Mφs can adopt particular phenotypes based upon various factors, including tissue-specific, context-dependent external cues received from their surrounding environment. In depth discussion of Mφ phenotypes, under physiological conditions and in cancer, are out with the scope of this review, and have been extensively reviewed previously (Wynn et al., 2013). In brief, Mφs can be broadly classified into two polar opposite phenotypes M1 and M2. At one extreme there are classical activated/inflammatory M1 Mφs expressing the antigen presentation molecules major histocompatibility complex class II (MHC-II), CD80, and CD86 (Figure 2). Mφs adopt this phenotype *via* the actions of interferon gamma (IFN-γ), granulocyte-macrophage colony-stimulating factor (GM-CSF) and lipopolysaccharide (LPS), which signal *via* signal transducer and activator of transcription (STAT) 1 and 2 (STAT1/STAT2) (Chávez-Galán et al., 2015). Furthermore, at the gene and protein level M1 Mφs express and release the pro-inflammatory mediators: nitric oxide synthase 2 (NOS2), IL-12, nitric oxide (NO), IL-1β, C-X-C motif ligand 10 (CXCL10) and tumour necrosis factor-α (TNF-α), exhibiting anti-leukaemia and immunostimulatory functions, and importantly enhancing cancer drug sensitivity (Yuan et al., 2015). At the other extreme lie alternatively activated/anti-inflammatory M2 Mφs, expressing the haemoglobin/haptoglobin and mannose scavenging receptors CD163 and CD206 (MRC1), respectively, as well as M-CSF receptor (M-CSFR/CD115). This phenotype is acquired *via* the actions of M-CSF, and the Th2 cytokines, IL-10, IL-4/IL-13, which signal *via* MYC/FOS, STAT3, STAT6, and C/EBPβ, respectively. Additionally, at the transcript and protein level these Mφ subpopulations express and secrete the enzymes arginase II (Arg2) and chitinase-3-like protein 1 (CHI3L1/YKL-40), and the anti-inflammatory cytokines IL-10 and TGF-β displaying leukaemia supporting and immunosuppressive properties (Mantovani and Allavena, 2015), as well as reducing cancer drug sensitivity (De Palma and Lewis, 2011, 2013; Figure 2). To add another layer of complexity, specific Mφs, known as tumour associated Mφs (TAMs) in solid cancers, or leukaemia associated macrophages (LAMs) with regards to leukaemia, have been described. These Mφ populations overlap functionally with M1 and M2-like Mφs; with M2-like Mφs predominantly exhibiting cancer-promoting functions, including cancer cell proliferation and dissemination. Infiltration of Mφs at cancer sites also correlates with therapy resistance, and inferior patient outcomes in many blood and solid cancers (Zhang et al., 2012; Mantovani et al., 2017; Wang et al., 2019). These Mφs display specific metabolic signatures/profiles similar to steady state M1 and M2-like Mφs (Caux et al., 2016; Vitale et al., 2019).

## AML-Driven Macrophage Phenotypic Reprogramming

There is mounting evidence demonstrating that *via* cell-to-cell contact, AML blasts have the capacity to re-educate monocytes and Mφs towards an M2-like leukaemia-supporting phenotype. This occurs through secreted factors and signal transduction modulation of transcription factors. Using *in vitro* and *in vivo* models, Mussai et al. (2013) provided the first reports showing that arginase II secreted from primary AML blasts re-educates healthy donor derived monocytes towards an M2-like phenotype, as demonstrated by an upregulation of CD206. Additionally, utilising primary xenograft models of AML, they demonstrated that primary AML blasts that successfully engrafted into non-obese diabetic/severe combined immunodeficiency (NOD-SCID) mice upregulated YM-1 (CHI3L1) on Ly6C^+^ BM monocytes. Importantly, *via* immunohistochemical analysis of BM samples from AML patients, they also showed high expression of CD206^+^ cells and arginase II. Providing further evidence that AML blasts have the ability to re-educate Mφs to a leukaemia supporting phenotype, Al-Matary et al. (2016), conducted pre-clinical studies with C1498-GFP, MLL-AF9, AML-ETO9a, and NUP98-HOXD13 syngeneic and transgenic AML mouse models. They showed that M2-like/alternatively activated Mφs (AAMs), characterised as CD11b^+^Ly6G^–^MHCII^–^Ly6C^–^ (Table 1), were elevated in the BM of leukaemic mice vs. non-leukaemic mice. Furthermore, BM derived Mφs (BMDMs) isolated from C1498-leukaemic mice, and co-cultured directly with C1498 murine AML cells, were shown to be more efficient at supporting the proliferation of the C1498 AML cells, compared to BMDMs isolated from non-leukaemic mice. Importantly, utilising the NUP98-HOXD13 mouse model, that mirrors the rare t(2;11)(q31;p15) translocation correlating with human myeloid malignancies (Lin et al., 2005), the authors established that the higher percentage of AAMs in these mice negatively correlated with their survival.

Mechanistic insights into the leukaemia-driven AAM phenotype in the leukaemic mice, was provided through transcription factor growth factor independence 1 (*Gfi1*) expression, essential for myeloid differentiation, and shown to be involved in the ability of AML cells to preferentially polarise Mφs to an M2-like phenotype, at the expense of M1-like Mφs. The authors showed that BMDMs derived from *Gfi1*-KO mice were enriched for M1-like vs. M2-like Mφs, when exposed to M1 and M2-polarising regimes, as determined by an increase in Ly6C^–^CD206^–^ cells (Table 1) exhibiting higher expression/secretion of the M1 markers, NOS2 and IL-6. Moreover, knock down of *Gfi1* provided a survival advantage to MLL-AF9 and NUP98-HOXD13 leukaemic mice, suggesting that targeting of *Gfi1*, could represent a new therapeutic strategy to inhibit stroma-driven disease mechanisms in AML. In support of AML blasts possessing the ability to downregulate M1-like Mφs, Keech et al. (2017) showed that following the establishment of moderate to high levels of leukaemia burden in MLL-AF9 AML mice, non-malignant/AML BM Mφs displayed a reduction in M1 Mφ markers, including MHC class II and CD80. On the contrary, previous observations by Yang et al. (2017) suggest that LAMs (designated by the authors as CD3^–^GR1^*low*^MCSFR^*int*^F4/80^*hi*^SSC^*low*^), isolated from the BM of secondary recipient mice, originally from MSCV-MLL-AF9-IRES-GFP AML mice, are enriched for M1 gene signatures, including iNOS, TNF, IL-6, and IL-1 (Table 1). Although the studies utilised the MLL-AF9 genetic murine model of AML, discrepancies between findings could be due to the authors focusing on potentially phenotypically different Mφ subsets i.e., Keech et al. (2017) centring on CD11b^–^F4/80^+^CD169^+^VCAM1^+^ and Yang et al. (2017) focusing their attention on CD3^–^GR1^*low*^MCSFR^*int*^F4/80^*hi*^SSC^*low*^ Mφs (Table 1). Furthermore, Keech et al. (2017) state that at 2-days post treatment with doxorubicin and cytarabine, a particular subset of Mφs (CD11b^–^F4/80^+^CD169^+^VCAM1^+^) persists within these AML mice. Discrepancies in the Mφ subsets identified could be due to key factors, such as disease severity/level of disease and time to analysis. Keech et al. (2017) unfortunately did not report on the AML disease burden in the MLL-AF9 mice following treatment with doxorubicin and cytarabine, and thus it is not possible to correlate Mφ phenotype to disease levels. Timing is important also, as during the early stages of cancer development M1-like anti-tumour Mφs are thought to infiltrate tumours. This is followed by their subsequent polarisation, along with myeloid-derived suppressor cells (MDSCs), into pro-tumour M2-like Mφs later in the course of the disease. This M1 to M2 switch is through sustained exposure to polarising factors released by the cancer cells and direct cell-to-cell contact between cancer cells and Mφs (Jackute et al., 2018).

The MLL-AF9 model is an aggressive murine AML model, and it is possible that if Mφs were analysed at different stages of leukaemia burden, this would affect the polarisation and proportion of different Mφ phenotypes. It is also possible that exposure to chemotherapeutics is enriching for specific Mφ subsets. Study findings by Nakasone et al. (2012) demonstrate that in a murine model of breast cancer, treatment with doxorubicin led to an enhanced recruitment of inflammatory CCR2^+^ monocytes, thought to be precursors of M2-like Mφs (Brown et al., 2017). It has also been recently reported that the interplay between epigenetic and microRNA/transcriptional factors drive impaired M1-like Mφ polarisation in AML, in which the dynamic interplay between the newly discovered histone acetyltransferase monocytic leukaemia zinc finger (*MOZ*), and the general myeloid regulator microRNA *miR-223* were inferred to be involved (Jiang et al., 2019). Treatment of murine BMDMs with the M1-polarising Toll-like receptor 4 ligand LPS decreased *MOZ* levels, with the converse observed when BMDMs were treated with the M2-polarising cytokine IL-4. Moreover, *MOZ* knock down in a murine Mφ cell line ameliorated LPS-driven M1-activation signatures, as evidenced by diminished IL-6 and TNF-α expression and a concomitant increase in the M2-associated marker IL-10, at both the transcript and protein level.

In AML patients, *MOZ* levels were shown to be reduced with a reciprocal elevation in the MOZ target *miR-223* as compared to healthy counterparts. Subsequent studies showed that human monocyte-derived macrophages (MDMs) displayed higher MOZ and lower *pri-miR*-223 expression following exposure to LPS with the opposite observed after IL-4 treatment. Importantly, low levels of *MOZ* were demonstrated to be present in the M5 monocytic AML FAB subtype, correlating with inferior overall survival (OS) in retrospective data collected from the cancer genome atlas (TCGA) cohort (Jiang et al., 2019). Findings from these studies suggest that AML blasts have the ability to induce phenotypic switching of Mφs towards a leukaemia supporting phenotype, at the expense of M1-like Mφs. Also, it is possible that distinct Mφ subsets could exhibit the capacity to support AML blasts, with exposure to chemotherapeutics potentially selecting for leukaemia supporting Mφs within the BMME. It is tempting to speculate that although LAMs display/share some characteristics of M2-like Mφs, they are likely to be more distinct from M2-like Mφs, and could be disease subset specific, especially given the complexity and heterogeneity of AML. Consequently, more in-depth analysis is warranted to fully determine LAM phenotypes within different subclasses of AML.

## Impact of M2-Like Macrophages on Therapy Response

**FIGURE 3:**
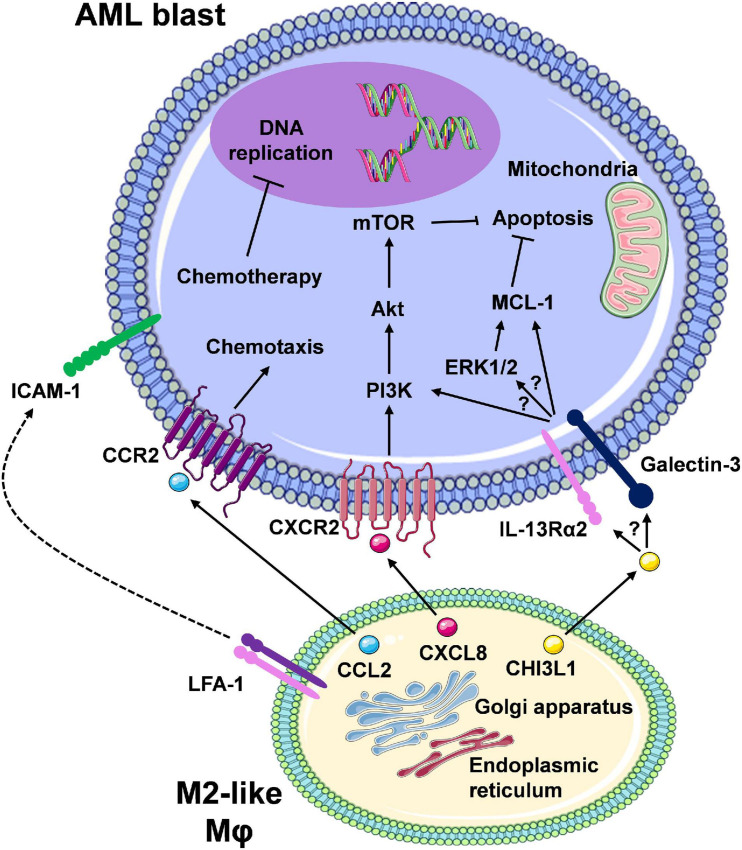
The interaction between M2-like macrophages and leukaemic cells promotes pro-survival/anti-apoptotic signalling in AML cells. Here, we are highlighting Mφ-AML blast interactions that mediate reduced therapeutic response and therapy resistance. Secretion of soluble factors (e.g., CCL2, CXCL8/IL-8) from Mφs *via* the ER/Golgi secretory pathway results in a number of downstream signalling events in the leukaemic blasts, many of which remain to be clearly elucidated (CCL2/CCR2, CXCL8/CXCR2, and speculated involvement of the CHI3L1/IL-13Rα2/Galectin axis). Overall, the net effect is the activation of pro-survival pathways (e.g., PI3K/Akt/mTOR, ERK1/2/MCL-1) and inhibition of apoptosis. Direct cell-to-cell contact, for example *via* ICAM-1-LFA-1 integrin interaction, may also result in similar intrinsic leukaemia cell responses. In turn, chemotherapy-mediated response (e.g., DNA damage) and apoptosis is prevented, which displays as chemotherapy resistance. Despite the development of targeted therapies for combination regimens with chemotherapy or as single agents, therapy resistance still remains a major limiting factor in patient outcomes due to these cell-to-cell interactions (created using Servier Medical Art).

The role of Mφs in treatment resistance in AML has not been fully elucidated. Initial insights suggest that AML-Mφ cross-talk occurs *via* direct Mφ-to-AML cell contact and/or Mφ secreted factors, thereby reducing drug sensitivity in AML cells. This is driven by the ability of Mφs to upregulate pro-survival/anti-apoptotic pathways in AML cells (Figure 3). These findings come from initial studies utilising murine models of AML, and from our own studies centring on the *in vitro* co-culture of primary monocyte-derived Mφs with AML cell lines (Williams et al., 2020). Focusing on a murine model of MLL-AF9 driven AML, preliminary findings from Keech et al. (2017) demonstrate that specific depletion of CD169^+^/SIGLEC1^+^ Mφs (Table 1), using heterozygous diphtheria toxin (DT) receptor expressing mice to selectively deplete host CD169^+^ Mφs *via* DT injection, significantly enhanced the median survival of mice treated with cytarabine and doxorubicin, as compared to AML counterparts exhibiting normal levels of BM CD169^+^ Mφs. Interestingly, depletion of CD169^+^ Mφs in the chemotherapy treated mice showed reduced extramedullary leukaemic burden in the blood and the spleen. However, the authors did not state the extent of CD169^+^ Mφ depletion in these mice, or specifically mention any changes in AML blast levels in the BM. Importantly, CD169^+^ TAMs have recently been identified in breast cancer patients, correlating with inferior disease-free survival (Cassetta et al., 2019). Interestingly in the healthy BM niche, CD169-expressing Mφs have been shown to function in retaining HSPCs (Chow et al., 2011). This raises the possibility that Mφs could provide supportive interactions for LSPCs. This is of particular importance, as LSPCs are often resistant to chemotherapy and represent the major source of AML relapse (Pabst et al., 2014). It is also possible that other non-CD169^+^ Mφs could be important for the support of AML blasts, specifically within the BM niche. Preliminary observations from our own studies, using M-CSF driven primary monocyte derived CD163^+^CD206^+^ Mφs (Table 1) (MDMs) from healthy donors, suggest that M2-like Mφs protect U937 and THP-1 AML cell lines from daunorubicin-induced apoptosis (Williams et al., 2020). Chemoresistance occurred *via* cell-to-cell contact, and to a certain extent through Mφ secreted factors. Furthermore, we have shown that Mφ secreted factors activate the extracellular signal-regulated kinases 1 and 2 (ERK1/2) pathway and upregulate MCL-1 protein expression (Williams et al., 2020). It is tempting to speculate that M2-like Mφs retain AML blasts within the BM *via* CCL2-CCR2 interactions.

In particular, M2-like Mφs constitutively release high levels of CCL2 (Sierra-Filardi et al., 2014), and AML blasts have been shown to exhibit CCL2-mediated migration by expression of functional CCR2 (Cignetti et al., 2003). Furthermore, in the vast majority of solid cancer models, M2-like Mφs and/or TAMs at the cancer site differentiate *in situ* from circulating CCR2^+^CD14^++^CD16^–^ classical monocytes (Table 1), recruited *via* cancer cell-secreted CCL2 (Qian et al., 2011; Cassetta et al., 2019). In support of this, initial studies by Jacamo et al. (2015) utilising the syngeneic AML1/ETO9a-expressing primary murine leukaemia model, showed that the selective blockade of CCR2 *via* the ribonucleic acid aptamer mNOX-E36, reduced the influx of CD11b^+^Ly6C^*low*^MHCII^*low*^ M2-like Mφs (Table 1) into leukaemia bearing organs, such as the spleen. Moreover, the level of CCL2 is a prognostic marker for AML, with higher serum levels of this chemokine detected in untreated AML individuals possessing unfavourable cytogenetics when compared to patients with favourable cytogenetics (Mazur et al., 2007). High CCL2 serum levels are also predictive of poor clinical outcomes in AML patients (Merle et al., 2019). Elevated serum levels of CHI3L1, in newly diagnosed AML patients, have also been associated with inferior survival at 1 and 12 months post diagnosis (Bergmann et al., 2005). CHI3L1 has been shown to induce ERK1/2 pathway activation (He et al., 2013), and contributes towards chemoresistance in ovarian cancer, *via* upregulation of MCL-1 protein levels (Chiang et al., 2015). In line with our aforementioned results (Williams et al., 2020), a colorectal cancer (CRC) focused study demonstrated that factors released by M2 polarised Mφs activate the ERK1/2-MCL-1 axis in immortalised and primary CRC cells (Lee et al., 2020). Interestingly from a therapeutic point of view, the authors showed that M1-like Mφs led to a reduction in ERK1/2-MCL-1 pathway activation. These observations suggest a possible link between Mφ induced activation of ERK1/2 and positive regulation of MCL-1 levels, potentially through the ability of ERK1 to stabilise MCL-1 protein levels, *via* phosphorylation of Threonine 163 within the regulatory Proline-Glutamate-Serine-Threonine (PEST) region of MCL-1 (Thomas et al., 2010).

Taken together these studies suggest that Mφs potentially limit the response of AML blasts towards conventional chemotherapeutics, *via* activation of pro-survival pathways in the latter. This is in agreement with well-established literature focusing on other haematological malignancies including chronic lymphocytic leukaemia (van Attekum et al., 2017) and multiple myeloma (Zheng et al., 2009; Beider et al., 2014). These findings therefore suggest that therapeutic strategies targeting M2-like Mφs/TAMs could potentially enhance drug sensitivity, subsequently improving clinical outcomes in AML patients.

## Clinical Implications and Potential Significance of Macrophages

Findings from recent studies show that the number of M2 and M1-like Mφs in the BM correlates with important patient outcomes, providing prognostic value in AML patients. Utilising the algorithmic program CIBERSORT, Xu et al. (2019) performed immunophenotypic analysis on datasets retrieved from the gene expression omnibus (GEO) and TCGA platforms, and demonstrated that there was an increased level of M2-like Mφs (defined by the authors as CD206^+^ cells) in the BM of AML patients compared to normal BM counterparts. Furthermore, M2-like Mφs represented a major component of the BMME, compared to other haematological malignancies, including chronic myeloid leukaemia. M2-like Mφ enrichment in AML patients, was confirmed through the algorithm, xCell, *via* data from two additional independent AML patient cohorts. Cognizant that in solid cancers the frequencies/levels of M2-like Mφs can predict clinical outcomes, the authors then conducted CIBERSORT analysis of two independent AML patient cohorts with available survival data. In one dataset (cohort 2; GSE10358, which included 304 AML patients, of which 188 AML patients were diagnosed with *de novo* AML, and exhibited somatic mutations in FLT3, KIT, and JAK2 tyrosine kinase, analysed by high-throughput re-sequencing), increased levels of M2-like Mφs correlated with inferior OS and event-free survival in AML patients, which they validated by xCell analysis.

This is in line with Yang et al. (2017)’s results, showing decreased probability of survival in AML patients with high CD163 transcript levels (as well as CD206, CD163 also used to define M2-like Mφs), as compared to patients with lower CD163 levels. Further confirming the association of CD163 with poor clinical outcomes, Guo et al. (2021) described the association of a distinct monocyte/macrophage cluster by single-cell RNAseq, highly expressing CD163, with reduced probability of survival in AML patients (Guo et al., 2021). Interestingly, Xu et al. (2019) also showed that the frequency of M1-like Mφs, in cohort 3 (GSE6891, this study included 461 blood and bone marrow samples from AML patients, under the age of 60, with gene expression analysed *via* an Affymetrix GeneChip^TM^ Human Genome U133 Plus 2.0 Array), arose as the sole and significant prognosticator for prolonged survival in AML patients. These findings indicate the ratio of M2-like to M1-like Mφs (M2/M1) could be of clinical importance to AML patients, as Chen et al. (2017) demonstrated that multiple myeloma patients with a high M2/M1 ratio, exhibited lower initial response rates to induction therapy, progression-free survival, and OS. Furthermore, there is a well-established body of evidence demonstrating that M2/M1 ratio has prognostic significance in a number of solid malignancies, including CRC (Lee et al., 2020). Analysing gene expression and clinical data from the Beat AML trial, Xu et al. (2019) then demonstrated that AML patients exhibiting higher CD206 expression levels had an inferior response to induction therapy compared to patients with a lower expression of CD206. Furthermore, patients in complete remission had significantly lower CD206 expression than patients exhibiting refractory (treatment resistant) disease. These findings are in line with reports from Keech et al. (2017), providing further evidence for the capacity of M2-like Mφs to potentially drive chemotherapy resistance in AML.

Xu et al. (2019) also showed that elevated expression of CD206 positively correlated with: morphological (FAB) subtypes M0 (undifferentiated AML—uncommon, usually associated with unfavourable risk) and M4 (myelomonocytic AML—common and intermediate risk); cytogenetic abnormality Inv (16) (normally linked with favourable risk); and negatively correlated with patients exhibiting the gene mutations *NPM1* and *IDH1*. A potential caveat to their study is that significant correlations were shown between inactive/resting dendritic cells (DCs) and CD206 expression. Therefore, the authors could not completely rule out the possible contribution of CD206^+^ DCs in their study. Complementing this work, is the study by Guo et al. (2021), describing the enrichment of two potentially immunosuppressive DC populations in AML BM, compared to a healthy BM. These were shown to be CD206^+^ DCs, associated with recruitment of regulatory T-cell populations, and T-cell suppressive CX3CR1^+^ DCs. This study highlights the power of single-cell sequencing in resolving and better understanding the complex immunological composition of the BMME of individual AML patients, and importantly how this information could potentially be utilised, to select patient-specific treatments targeting resident immune cell populations. Furthermore, this study highlights the importance of distinguishing CD206^+^ macrophages from CD206^+^ DCs, as these different immune cell subtypes may have different impacts on clinical outcomes in AML patients. Another consideration not fully recognised by Xu et al. (2019) is that in some cases of AML, malignant differentiated monocyte-like cells also express high levels of CD206, previously demonstrated to correlate with reduced survival in AML patients (van Galen et al., 2019).

Despite the observation from another study demonstrating that CD163^+^ and CD206^+^ macrophage populations were found to be infrequently enriched in AML BM samples, analysis at the single cell level will aid in identifying rare cell types that, when found alone or in association with other immune cell types, may exacerbate disease pathogenesis and therefore negatively impact patient prognosis (Guo et al., 2021). Consequently, further studies are required to fully ascertain the contribution of both *bona fide* non-malignant, as well as malignant/AML M2 and M1-like monocytes/Mφs in AML pathogenesis, and to determine if the M1/M2 ratio has potential as a prognosticator for outcome and treatment response in AML.

## Therapeutic Targeting of M2-Like Macrophages in AML

Numerous therapeutic strategies have been devised to target M2-like Mφs/TAMs. These include: inhibiting the recruitment of CCR2^+^CD14^++^CD16^–^ monocytes (TAM precursors), *via* CCL2-CCR2 blockade; specific deletion/depletion *via* anti-CD115/M-CSFR antibodies; and phenotypic/functional reprogramming to M1-like anti-leukaemia Mφs, through DICER inhibition-mediated upregulation of IFN-γ/STAT1-inducible genes and modulation of microRNAs. An overview of TAM targeting strategies are eloquently discussed in recent review articles (Cassetta and Pollard, 2018; Anfray et al., 2020; Lopez-Yrigoyen et al., 2020). Deciphering distinct macrophage populations within individual patients will be of paramount importance to guide/inform personalised treatment options, for targeting one or more immune populations alongside the main cancerous cell population (Arlauckas et al., 2021). PI3K isoform-selective inhibitors are currently in pre-clinical development and/or early phase clinical trials and hold much promise for AML patients. Not only do they have the potential to suppress/inhibit Mφ infiltration into tumours and re-educate Mφs to an M1-like phenotype, but they also display direct killing effects on AML blasts.

It is well-established that the pro-survival PI3K-Akt-mTOR axis is abnormally upregulated in AML patients, through various molecular mutations (e.g., receptor tyrosine kinases, GTPases, *FLT3-*ITD) (Kandoth et al., 2013; Weinstein et al., 2013), with constitutive induction of this pathway occurring on average in almost two thirds of AML patients, and correlating with inferior survival (Min et al., 2003; Xu et al., 2003; Kornblau et al., 2009; Chen et al., 2010; Nepstad et al., 2019, 2020). The Class I PI3K are composed of the regulatory p85 subunit and catalytic p110 subunits, in which the latter can be sub-divided into 4 isoforms; α, β, γ, and δ. In murine models of breast and lung cancer, genetic knock out of PI3Kγ reinstructed M2-like TAMs towards an M1-like Mφ phenotype, exhibiting enhanced surface expression of MHC II (M1 marker). Tumour cells and TAMs also exhibited increased *Nos2*, *IL-12b*, and *IFN*-γ expression with a concomitant reduction in the expression of the M2- Mφ markers *arg1*, *IL-10*, *TGF*-β (Kaneda et al., 2016). However, the authors noted that PI3Kγ KO did not prevent TAMs (CD11b^+^Gr1^–^F4/80^+^) from amassing in the tumours (Table 1). In agreement with these findings, a recent study investigating the impact of the PI3Kγ/δ inhibitor TG100-115 on CRC, showed enhanced expression of *IL*-1β and *CXCL10*, as well as reduced *IL-10* and *TGF*-β in tumours from TG100-115 treated mice (Lee et al., 2020). In contrast to Kaneda et al. (2016), the authors showed that PI3K knock down induced M1-like (MHC II^+^) Mφ infiltration, while suppressing M2-like (CD206^+^) Mφ infiltration into tumours. Reduced TAM infiltration likely resulted from the inhibition of PI3Kγ-dependant migration signals, that have been documented to occur through G-protein coupled receptor induction in Mφs and their precursors (O’Hayre et al., 2014). The reported discrepancies could be related to differences in the model systems studied i.e., breast and lung cancer model vs. CRC murine model and genetic KO of PI3Kγ, compared to pharmacological inhibition of both PI3Kγ and PI3Kδ. Aforementioned, PI3K isoform-selective inhibitors also have the additional benefit of demonstrating direct cytostatic and cytotoxic effects on AML blasts.

Studies to date have reported that AML blasts always express p110δ, whereas the incidence of p110γ expression and other isoforms, are highly variable, owing to the heterogenic molecular landscape observed in AML (Billottet et al., 2006; Tamburini et al., 2007).

Dual targeting of PI3Kγ and PI3Kδ *via* the selective inhibitor IPI-145/duvelisib, has been demonstrated to decrease survival and induce cellular apoptosis in AML cell lines and primary AML blasts (Pillinger et al., 2016). This observation was likely the result of reduced activation of the Akt and ERK pro-survival pathways in the AML cells, as shown by the authors in subsequent studies. Furthermore, duvelisib may also have additional benefit, with its potential to overcome stroma-mediated resistance, as it was shown to reduce primary AML blast adhesion to primary BMSCs, and subsequently re-sensitise primary AML blasts to cytarabine and daunorubicin when co-cultured with BMSCs. Disappointingly, findings from a phase I clinical trial, failed to demonstrate any therapeutic value of duvelisib in the AML patients studied (Flinn et al., 2018). It is important to note, however, that only a small AML patient cohort (*n* = 6) was included in this initial trial, and AML patients were treated only with the 75 mg dose, and not at the higher 100 mg dose. Moreover, the cytogenetic and mutational status of the patients are not provided, and it is uncertain whether treatment naïve (TN) or relapsed/refractory (R/R) AML patients were included in this study. This has now been extended to a Phase II trial (NCT02711852, from Supplementary Table 1), to assess long-term safety of duvelisib monotherapy in CR patients with haematological malignancies previously enrolled in the phase I trial. Moving forward, a larger-scale clinical trial involving TN and R/R AML patients, representing different cytogenetic and mutational subtypes (e.g., FLT3-ITD^+^ patients with constitutive PI3K pathway activation), is necessary to fully ascertain the therapeutic potential of duvelisib and other PI3K isoform-selective inhibitors of AML.

In addition to the aforementioned macrophage-targeting strategies, there is a substantial body of evidence suggesting that checkpoint inhibition at the CD47-SIRPα level, is a highly promising therapeutic approach for various cancers, including AML. Over a decade ago, two back-to back seminal research publications, demonstrated that the transmembrane and macrophage checkpoint protein CD47, was overexpressed on AML cell lines (Jaiswal et al., 2009), as well as primary bulk AML cells and AML LSPCs, as compared to non-AML counterparts (Majeti et al., 2009). Importantly, through analysis of microarray datasets from three independent adult AML patient cohorts (Valk et al., 2004; Bullinger et al., 2008; Metzeler et al., 2008), Majeti et al. (2009), demonstrated that high CD47 levels, were associated with a reduction in event-free survival and OS, as compared with AML patients exhibiting low expression of CD47. Furthermore, Jaiswal et al. (2009) showed that genetically induced overexpression of CD47 in the AML cell line MOLM-13, correlated with the capacity of these AML cells to escape macrophage-mediated elimination *via* phagocytosis. These observations, along with previous reports that CD47 binding to phagocyte/macrophage expressed signal regulatory protein alpha (SIRPα) negatively regulates phagocytosis (Brown and Frazier, 2001; Barclay and Brown, 2006), provided a clear rationale for blocking the interaction between CD47-SIRPα. This has subsequently led to considerable efforts in developing high affinity and specific anti-CD47 monoclonal antibodies (mAbs) and SIRPα-Fragment crystallisable (Fc) fusion proteins, which have been comprehensively discussed in a recent review article (Zhang et al., 2020). Although these agents have shown great potential as effective therapies in pre-clinical studies, they do possess some limitations and adverse reactions, associated with non-tumour targeting of the CD47-SIRPα axis. These adverse reactions include therapy-induced anaemia, due to erythrocytes/red blood cells (RBCs), and in particular aged RBCs, expressing high levels of CD47 on their surface. RBCs therefore act as antigen sinks, and with this in mind, researchers have generated modified anti-CD47 antibodies, which exhibit reduced RBC binding and enhanced specific binding to AML cells, either *via* silencing of the Fc region (Pietsch et al., 2017) or augmentation through the incorporation of CD33-targeting light chain (Ponce et al., 2017).

A particularly promising anti-CD47 mAb is the fully humanised antibody Hu5F9-G4/Magrolimab (Supplementary Table 1). Recent findings from a phase 1b/II study, involving 52 treatment-naïve AML patients with adverse cytogenetic and molecular aberrations (complex cytogenetics and TP53-mutated AML), that were given Magrolimab in combination with azacytidine (AZA), showed that almost half of TP53-mutant AML patients achieved complete remission, and a median OS of 12.9 months (Sallman et al., 2020). In addition, as Magrolimab has previously been shown to induce macrophage-mediated phagocytosis of AML LSCs (Majeti et al., 2009), the investigators assessed the levels of LSCs in the BM of AML patients following combination therapy, with these studies demonstrating that Magrolimab + AZA eliminated LSCs in almost three quarters of AML patients in the study. The authors also mention that a phase 3 study is planned.

## Conclusion and Perspectives

Studies investigating the role of Mφs in AML have been hindered to some extent, due to challenges surrounding the ability to accurately distinguish non-malignant *bona fide* Mφs from malignant/AML-Mφs. Most studies to date have employed conventional methodologies e.g., immunohistochemistry and flow cytometry, to assess M2-like Mφs/monocytes in the BM or spleen of AML patients and murine models, respectively, based upon myeloid markers (e.g., CD163 and CD206). However, these myeloid markers are now known to be expressed and shared by both non-malignant monocytes and Mφs and AML-Mφs. Encouragingly, the recent applications of single-cell RNA-seq and genetic profiling, have been able to detect differences in transcript expression and mutations within malignant AML cells, which are now allowing researchers to specifically distinguish and characterise non-malignant and malignant-Mφs within the AML BMME. Nevertheless, in comparison to the progress made in the understanding of M2-like Mφs/TAMs in solid malignancies, our basic understanding of Mφ biology in AML is still in its infancy. Many fundamental questions surrounding the frequency and composition of Mφ phenotypes within the BMME and their contributions to disease processes, such as therapy resistance and disease relapse/progression, have still to be addressed. With the advent of new enabling technologies, including multiplex imagining modalities (e.g., CO-Detection by indEXing (CODEX) platform) (Schürch et al., 2020), researchers will be able to visualise and determine cellular interactions between AML blasts and their surrounding BMME. For instance, use of antibodies directed against monocyte/Mφ markers (CD163, CD206, CD14), as well as antibodies directed against proteins frequently mutated in AML (e.g., NPM1c^+^ protein or FLT3), will enable researchers to distinguish and study the interaction of *bona fide* non-malignant M2-like NPM1c^–^/FLT3^–^CD163^+^CD206^+^ Mφs with NPM1c^+^/FLT3^+^CD163^+/–^CD206^+/–^ AML blasts in the BMME of NPM1-mutant and FLT3 AML patients, with the potential to apply this technology to other molecular subtypes of AML. Furthermore, we are currently in the profiling era of advanced technology, in which we can undertake profiling of the secretome using Luminex, SWATH proteomics, as well as scRNA sequencing and metabolomics, enabling us to understand AML pathogenesis at the single cell level.

With the advent of a recently developed macrophage annotation platform, MacSpectrum, which draws upon single-cell RNA-seq data, and has demonstrated the ability to resolve distinct and heterogeneous BM-derived and adipose tissue-derived macrophage populations (Li C. et al., 2019), it is clear that single-cell sequencing is at the forefront of distinguishing heterogeneous macrophage subpopulations from DCs, and is thus catalysing our understanding of the complex and diverse role of macrophages in various disease settings, including AML.

It is crucial that LAMs and tissue-resident Mφs in the BMME of AML patients, are fully characterised at the phenotypic and functional level, in order to determine molecular (transcript/protein) differences, that could be exploited therapeutically, for the specific targeting of LAMs. This will be paramount in the ability to generate future Mφ targeting strategies, exhibiting desirable pharmacological properties, such as enhanced efficacy/on-target effects, and reduced toxicity owing to off-target effects. Given that Mφ-mediated therapy resistance likely occurs *via* the upregulation of pro-survival/anti-apoptotic pathways, such as ERK1/2 and MCL-1 in AML cells, treatment combinations comprising selumetinib and/or AZD5991 or CYC065, could represent novel therapeutic strategies to overcome Mφ, and more specifically, MCL-1-driven therapy resistance in AML.

Finally, with the advent of new immune-oncology based companies established to develop first-in-class compounds to target Mφ-mediated immunomodulation, the future holds much promise for the generation of novel therapeutic strategies, capable of safely and effectively modulating/targeting Mφ-driven disease processes in AML and other malignancies.

## Author Contributions

KM and MW wrote the manuscript. KM prepared the figures. KM, MW, MG, and HW contributed to the discussion of the draft. All authors contributed to the article and approved the submitted version.

## Conflict of Interest

The authors declare that the research was conducted in the absence of any commercial or financial relationships that could be construed as a potential conflict of interest.
