# Supplementary material for: Macrophages in Acute Myeloid Leukaemia: Significant Players in Therapy Resistance and Patient Outcomes
Source: Front Cell Dev Biol. 2021 Jun 24;9:692800. doi: 10.3389/fcell.2021.692800 (PMC8264427; doi:10.3389/fcell.2021.692800)
Supplement: Supplementary file 1 [file Table_1.DOCX]

**Supplementary Table 1**: Examples of ongoing clinical trials investigating targeted therapies as single agents and as combination regimens in adult acute myeloid leukaemia.

| **Drug** | **Mechanism of Action/Target** | **Purpose/Eligibility** | ***Clinical trial details** |
| --- | --- | --- | --- |
| *Targeted therapies/Combination therapies* | | | |
| Venetoclax | Selective BCL-2 inhibitor | Assessing the safety and efficacy of venetoclax in routine clinical practise for adult AML (≥18 years) | Study ID: NCT03987958  Phase N/A (Recruiting)  Estimated completion July 1, 2023 |
| Venetoclax, Alvocidib | Venetoclax: selective BCL-2 inhibitor  Alvocidib: CDK9 inhibitor | Assessing the safety, PK and preliminary efficacy of combination therapy in R/R adult AML (≥18 years) | Study ID: NCT03441555  Phase Ib  Completed January 25, 2021-Awaiting results |
| CYC065 (Fadraciclib), Venetoclax | CYC065 (Fadraciclib): dual MCL-1 and CDK2/5/9 inhibitor  Venetoclax: selective BCL-2 inhibitor | Assessing the safety and efficacy of combination therapy in R/R adult AML or MDS (≥18 years) | Study ID: NCT04017546  Phase I (Recruiting)  Estimated completion December 31, 2020 – No updates as of April 2021 |
| AZD5991, Venetoclax | AZD5991: selective  MCL-1 inhibitor  Venetoclax: selective BCL-2 inhibitor | Assessing the safety, tolerability, PK and preliminary anti-tumour activity of AZD5991 (± venetoclax) in R/R haematological malignancies (≥18 and ≤85 years) | Study ID: NCT03218683  Phase I/Ib/IIa (Recruiting)  Estimated completion November 30, 2022 |
| S64315, Venetoclax | S64315: selective MCL-1 inhibitor  Venetoclax: selective BCL-2 inhibitor | Assessing the safety, tolerability and recommended phase II dose of combination therapy in adult *de novo* AML, s-AML or t-AML (≥18 years) | Study ID: NCT03672695  Phase Ib (Recruiting)  Estimated completion September 30, 2022 |
| Ruxolitinib, Venetoclax | Ruxolitinib: JAK1/JAK2 inhibitor  Venetoclax: selective BCL-2 inhibitor | Assessing the safety and maximum-tolerated dose of combination therapy in adult R/R AML (≥18 years) | Study ID: NCT03874052  Phase I (Recruiting)  Estimated completion December 31, 2022 |
| Venetoclax, Azacitidine | Venetoclax: selective BCL-2 inhibitor Azacitidine: HMA | Assessing the efficacy of combination therapy in previously untreated elderly AML patients (≥60 years) | Study ID: NCT03466294 Phase II (Active, Not recruiting) Estimated completion October 2022 |
| Venetoclax, Decitabine | Venetoclax: selective BCL-2 inhibitor Decitabine: HMA | Assessing the safety and tolerability of combination therapy in adult AML patients (≥18 years) | Study ID: NCT03844815 Phase I (Not yet recruiting) Estimated completion June 10, 2024 |
| HDM201,  MBG453, Venetoclax | HDM201: selective p53-Mdm2 interaction inhibitor  MBG453: anti-Tim3 antibody  Venetoclax: selective BCL-2 inhibitor | Assessing the safety, tolerability, PK, PD and preliminary anti-tumour activity of HDM201 in combination with either MBG453 or venetoclax in adult AML or high-risk MDS patients (≥18 years) | Study ID: NCT03940352 Phase Ib (Recruiting) Estimated completion December 29, 2021 |
| Duvelisib | PI3Kγ/δ inhibitor | Assessing the long-term safety of duvelisib in adult patients with haematological malignancy previously treated with the compound (≥18 years) | Study ID: NCT02711852  Phase II Completed May 4, 2020-Awaiting results |
| AZD4573 | Selective CDK9 inhibitor | Assessing the safety, tolerability, PK, PD and preliminary anti-tumour activity of AZD4573 in adults with R/R haematological malignancies (≥18 years) | Study ID: NCT03263637 Phase I (Recruiting) Estimated completion December 31, 2021 |
| LY3214996 | ERK inhibitor | Assessing the safety and defining the appropriate dose of the investigational drug LY3214996 in R/R adult AML (≥18 years) | Study ID: NCT04081259 Phase I (Recruiting) Estimated completion April 30, 2023 |
| ASTX660,  ASTX727 | ASTX660: dual cIAP and XIAP inhibitor  ASTX727: fixed-dose combination of the HMA decitabine and the cytidine deaminase inhibitor cedazuridine | Assessing the safety, PK and efficacy of ASTX660 ± ASTX727 in R/R adult AML (≥18 years) | Study ID: NCT04155580  Phase I (Recruiting)  Estimated completion October 2021 |
| IBI188, Azacitidine | IBI188: anti-CD47 mAb Azacitidine: HMA | Assessing the safety, tolerability and composite CR of combination therapy in adult AML (≥18 years) | Study ID: NCT04485052 Phase Ib/II (Recruiting) Estimated completion May 2022 |
| Glasdegib,  Decitabine | Glasdegib: Hedgehog inhibitor  Decitabine: HMA | Assessing the response rates (CR/CRi) of combination therapy in patients with newly diagnosed poor-risk AML (≥18 years) | Study ID: NCT04051996  Phase II (Active, Not recruiting)  Estimated completion September 15, 2022 |
| Ruxolitinib,  Fedratinib, Decitabine | Ruxolitinib: JAK1/JAK2 inhibitor  Fedratinib: selective JAK2 inhibitor Decitabine: HMA | Assessing the efficacy of decitabine in combination with ruxolitinib or fedratinib before HSCT in AML originating from MPN (accelerated/blast phase MPN) (≥18 years) | Study ID: NCT04282187 Phase II (Recruiting) Estimated completion November 11, 2024 |
| *Antibody therapies/Combination therapies* | | | |
| Tocilizumab | Anti-IL-6Rα mAb | Assessing the addition of tocilizumab to standard induction chemotherapy (idarubicin + cytarabine) in high-risk adult AML (≥18 years) | Study ID: NCT04547062 Phase I (Recruiting) Estimated completion June 15, 2023 |
| Ipilimumab,  Nivolumab | Ipilimumab: anti-CTLA-4 mAb Nivolumab: anti-PD-1 mAb | Assessing the best dose and side effects of either ipilimumab or nivolumab in relapsed, adult haematologic cancers following allogeneic-HSCT (≥18 years) | Study ID: NCT01822509 Phase I/Ib (Active, Not recruiting) Estimated completion April 1, 2022 |
| Camrelizumab, Decitabine | Camrelizumab: anti-PD-1 mAb  Decitabine: HMA | Assessing the safety and efficacy of combination therapy in R/R elderly AML patients (60-75 years) | Study ID:  NCT04353479  Phase II (Not yet recruiting)  Estimated completion December 31, 2022 |
| Cusatuzumab,  Venetoclax, Azacitidine | Cusatuzumab: anti-CD70 mAb Venetoclax: selective BCL-2 inhibitor  Azacitidine: HMA | Assessing the safety and tolerability of cusatuzumab in combination with venetoclax ± azacitidine (≥18 years) | Study ID: NCT04150887 Phase Ib (Recruiting) Estimated completion June 30, 2023 |
| Magrolimab, Venetoclax, Azacitidine | Magrolimab: anti-CD47 mAb Venetoclax: selective BCL-2 inhibitor  Azacitidine: HMA | Assessing the side effects and best dose of magrolimab in combination with venetoclax and azacitidine (≥18 years) | Study ID: NCT04435691  Phase Ib/II (Recruiting) Estimated completion December 31, 2021 |
| Pembrolizumab,  Venetoclax,  Azacitidine | Pembrolizumab: anti-PD-1 mAb Venetoclax: selective BCL-2 inhibitor Azacitidine: HMA | Assessing the response of adult patients with newly diagnosed AML to venetoclax + azacitidine combination therapy ± pembrolizumab (≥60 years, ineligible for conventional chemotherapy) | Study ID: NCT04284787 Phase II (Recruiting) Estimated completion August 1, 2021 |
| PF-04518600, GO, Avelumab,  Azacitidine,  Glasdegib (maleate), Venetoclax | PF-04518600: anti-OX40 (CD134) antibody  GO: anti-CD33  Avelumab: anti-PD-L1 immunotherapy  Azacitidine: HMA  Glasdegib: Hedgehog inhibitor  Venetoclax: selective BCL-2 inhibitor | Assessing the side effects and best dose of PF-04518600 monotherapy and its combination with GO, avelumab, azacitidine, glasdegib and venetoclax in R/R AML (≥18 years)  This study includes 6 experimental treatment arms (A-F) ± PF-04518600 | Study ID: NCT03390296  Phase Ib/II (Recruiting) Estimated completion December 29, 2024 |
| *Standard induction chemotherapy/Combination therapies* | | | |
| CPX-351,  Glasdegib | CPX-351: 7+3 ratio of cytarabine and daunorubicin for prolonged release and anti-neoplastic effects  Glasdegib: Hedgehog inhibitor | Assessing the efficacy of combination therapy in previously untreated AML patients with MDS-related changes or t-AML (≥18 years) | Study ID:  NCT04231851  Phase II (Recruiting)  Estimated completion September 30, 2022 |
| Cytarabine, Daunorubicin, Gilteritinib,  Midostaurin | Cytarabine: anti-neoplastic agent  Daunorubicin: anti-neoplastic agent Gilteritinib: FTL3 inhibitor  Midostaurin: multi-targeted TKI | Assessing and comparing the efficacy of gilteritinib or midostaurin in combination with conventional chemotherapy in AML with FLT3 mutation (≥18 and ≤65 years) | Study ID: NCT03836209 Phase II (Recruiting) Estimated completion September 2024 |
| Cytarabine, Daunorubicin,  GO,  Midostaurin | Cytarabine: anti-neoplastic agent Daunorubicin: anti-neoplastic agent  GO: anti-CD33 mAb  Midostaurin: FTL3 inhibitor | Assessing the safety and tolerability of combination therapy in newly diagnosed FLT3-mutated adult AML (≥18 years) | Study ID:  NCT03900949  Phase I (Recruiting)  Estimated completion January 1, 2025 |
| Cytarabine,  Daunorubicin,  Idarubicin, Pembrolizumab, HSCT | Cytarabine: anti-neoplastic agent Daunorubicin: anti-neoplastic agent Idarubicin: anti-neoplastic agent Pembrolizumab: anti- PD-1 mAb | Assessing the response of adult patients with newly diagnosed AML, s-AML or AML arising from prior MDS to cytarabine plus daunorubicin or idarubicin ± pembrolizumab (≥18 and ≤75 years). Patients who achieve CR/CRi may undergo HSCT | Study ID:  NCT04214249 Phase II (Recruiting) Estimated completion July 31, 2024 |

| **Highlighted: potential for targeting of macrophages** |
| --- |

Abbreviations: AML, acute myeloid leukaemia; BCL-2, B-cell lymphoma 2; CDK, cyclin-dependent kinase; cIAP, cellular inhibitor of apoptosis; CR, complete remission; CRi, complete remission with incomplete blood count recovery; CTLA-4, cytotoxic T-lymphocyte-associated protein 4; ERK, extracellular regulated kinase; FLT3, FMS-like tyrosine kinase-3; GO, gemtuzumab ozogamicin; HMA, hypomethylating agent; HSCT, haematopoietic stem cell transplantation; IL-6Rα, interleukin-6 receptor alpha; JAK, Janus kinase; mAb, monoclonal antibody; MCL-1, myeloid cell leukaemia-1; Mdm2, mouse double minute 2 homolog; MDS, myelodysplastic syndrome; MPN, myeloproliferative neoplasms; PD, pharmacodynamics; PD-1, programmed cell death protein 1; PD-L1, programmed death-ligand 1; PI3Kγ/δ, phosphoinositide-3-kinase gamma/delta isoform; PK, pharmacokinetics; p53, tumour protein 53; R/R, relapsed/refractory; s-AML, secondary acute myeloid leukaemia; t-AML, therapy-related acute myeloid leukaemia; Tim3, T-cell immunoglobulin mucin-3; TKI, tyrosine kinase inhibitor; XIAP, X-linked inhibitor of apoptosis protein.

*All clinical trials are registered at clinicaltrials.gov and can be accessed by the Study ID provided in the table.

Information correct as of 04/04/2021.
